# The tumor-inhibitory effectiveness of a novel anti-Trop2 Fab conjugate in pancreatic cancer

**DOI:** 10.18632/oncotarget.8529

**Published:** 2016-04-01

**Authors:** Yuan Mao, Xiaoying Wang, Feng Zheng, Changjun Wang, Qi Tang, Xiaojun Tang, Ning Xu, Huiling Zhang, Dawei Zhang, Lin Xiong, Jie Liang, Jin Zhu

**Affiliations:** ^1^ Department of Oncology, Jiangsu Province Geriatric Hospital, Nanjing 210024, China; ^2^ Huadong Medical Institute of Biotechniques, Nanjing 210002, China; ^3^ Department of Pathology and The Key Laboratory of Antibody Technique of Ministry of Health, Nanjing Medical University, Nanjing 210029, China; ^4^ Department of Gynecology and Obstetrics, Nanjing Maternal and Children Care Hospital Affiliated to Nanjing Medical University, Nanjing 210029, China; ^5^ Department of Otolaryngology-Head and Neck Surgery, The Second Affiliated Hospital of Nanjing Medical University, Nanjing 210011, China; ^6^ Department of Pathology, The Second Affiliated Hospital of Nanjing Medical University, Nanjing 210011, China; ^7^ Department of Pathology, Wuxi Nanjing Maternal and Children Care Hospital Affiliated to Nanjing Medical University, Wuxi 214002, China

**Keywords:** Trop2, Fab, DOX, ADC, pancreatic carcinoma

## Abstract

Human trophoblastic cell surface antigen 2 (Trop2) has been reported to act oncogenically. In this study, one-step quantitative real-time polymerase chain reaction (qPCR) test and immunohistochemistry (IHC) analysis with were employed to evaluate the relationship between Trop2 expression and the clinicopathological features of patients with PC. Then a novel anti-Trop2 Fab antibody was conjugated with Doxorubicin (DOX) to form Trop2Fab-DOX, an antibody-drug conjugate. This Trop2Fab-DOX conjugate was characterized by cell ELISA and immunofluorescence assay. MTT and wound healing analyses were used to evaluate the inhibitory effect of Trop2Fab-DOX on PC cell growth *in vitro*, while xenograft nude mice model was established to examine the tumor-inhibitory effects of PC *in vivo*. High Trop2 expression was observed in PC tissues and Trop2 expression was associated with several malignant attributes of PC patients, including overall survival. Trop2Fab-DOX can bind to the Trop2-expressing PC cells and provide an improved releasing type of DOX. In addition, Trop2Fab-DOX inhibited the proliferation and suppressed the migration of PC cells in a dose-dependent manner *in vitro*, while inhibited the growth of PC xenografts *in vivo*. Trop2 is a specific marker for PC, and a novel Trop2Fab-DOX ADC has a potent antitumor activity

## INTRODUCTION

Pancreatic carcinoma (PC) is one of the most aggressive forms of digestive tract malignancies, and it ranks eighth in cancer-related mortality worldwide [1, 2]. The prognosis of patients with PC is extremely poor, with a 5-year survival rate of 1–4%, typically because of high lethality, delayed diagnosis, early metastasis, and severe progression [3, 4]. So far, conventional PC treatment merely relies on chemotherapy and radiotherapy. Although some novel approaches have been introduced for PC treatment in latest years, none can effectively improve its overall survival [5, 6]. It is thereby of timely importance to develop a novel treatment strategy that can predict the prognosis and improve therapeutic outcome in patients with PC.

Human trophoblastic cell surface antigen 2 (Trop2), also known as TACSTD2, EGP-1 or GA733-1, is a cell-surface glycoprotein, and belongs to the TACSTD gene family [7–9]. Trop2 expression was detected in a variety of human cancer cells, and its elevated expression is often associated with poor prognoses of breast, colon, and gastric cancers [10–12]. Moreover, the distribution of Trop2 is more differentiated in human cancers, suggestive of a certain oncogenic characteristic of Trop2 as a promising novel target for personalized treatment [13, 14].

Trop2 antibody has been constructed and tested by our former research, and human anti-Trop2 engineering antibody was used in immunohistochemistry (IHC) analysis [15] to investigate the inhibitory effectiveness of anti-Trop2 Fab antibody (Trop2Fab) in breast cancer [16]. For practical use, it is of added interest to motivate the application of Trop2Fab in cancer therapy with a more extended scale or circumstance, such as developing an antibody that can block the Trop2/MAPK pathway or conjugating an antibody with immunotoxins, radioimmunoconjugates or chemotherapy drugs [17].

Doxorubicin (DOX) is one of the most common chemotherapy drugs, and has been widely administrated in the clinical treatment for PC. Although patients under DOX therapy often experience serious side effects, such as heart failure, kidney toxicity and myelosuppression [18], DOX has some advantages for the research of antibody-drug conjugates, including high solubility, simple detectability and easy observability [19]. Recently, a number of drug molecules and delivery systems for DOX application have been developed with a promising prospect [20–22]. Based on the above, we hypothesized that a conjugate of Trop2Fab with DOX (Trop2Fab-DOX) could not only benefit from the high specificity of Trop2Fab fragment and but also reduce the possibility of side effects from DOX, as doing so can help characterize and optimize the clinical application of Trop2Fab.

To fill this void in knowledge, we first examined Trop2 expression in PC tissues and evaluated its relationship with the clinicopathological attributes of patients with PC. We next conjugated Trop2Fab with DOX to form an antibody-drug (Trop2Fab-DOX) conjugate. Further, we explored the antitumor effect of this Trop2Fab-DOX conjugate *in vitro* and *in vivo*.

## RESULTS

### Detection of Trop2 expression by qPCR and IHC analyses

**Figure 1:**
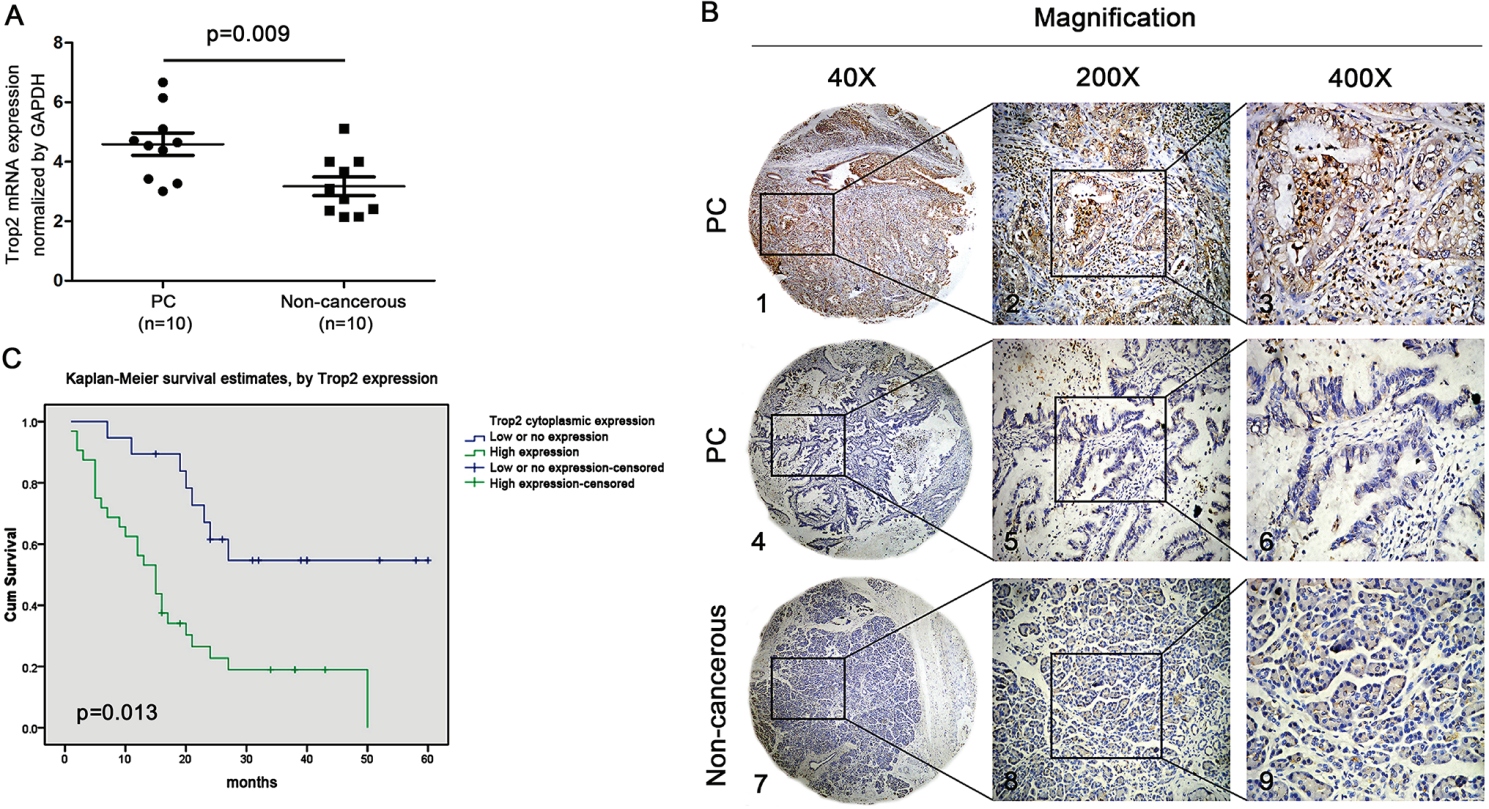
Trop2 expression in PC tissues and its relationship with survival status in patients with PC (**A**) Trop2 expression in pancreatic carcinoma (PC) tissues and non-cancerous tissues. One-step quantitative real-time polymerase chain reaction (qPCR) indicated that Trop2mRNA expression in PC tissues (4.6 ± 0.38) was significantly higher than that in non-cancerous tissues (3.2 ± 0.31) (*p = 0.009). (**B**) Representative images of Trop2 protein expression in PC and the corresponding non-cancerous tissues with tissue microarray (TMA) by immunohistochemistry (IHC) analysis. B1, B2 and B3 High staining of Trop2 in PC samples. B4, B5 and B6 Low staining of Trop2 in PC samples. B7, B8 and B9 Low staining of Trop2 in non-cancerous samples. Original magnification ×40 in B1, B4 and B7; ×200 in B2, B5 and B8; ×400 in B3, B6 and B9. (**C**) Kaplan-Meier curve demonstrated that overall survival rate in PC patients with high Trop2 expression (green line) was significantly lower than that in patients with low or no Trop2 expression (blue line).

For qPCR analysis, the expression of Trop2 mRNA was significantly higher in PC tissues than in the corresponding non-cancerous tissues when normalized to GAPDH (4.6 ± 0.38 vs. 3.2 ± 0.31, *p* = 0.009) (Figure 1A). For IHC analysis, high Trop2 expression in cytoplasm and stroma was respectively detected in 114 of 189 (60.3%) and 71 of 155 (45.8%) PC tissues. In comparison, high Trop2 expression in cytoplasm and stroma was only witnessed in 6 of 87 (6.90%) and 3 of 87 (3.44%) non-cancerous tissues. The differences of Trop2 expression (in both cytoplasm and stroma) between PC tissues and non-cancerous tissues were statistically significant (both *p* < 0.05).

The typical IHC staining for Trop2 expression and its relationship with important clinical characteristics in patients with PC are presented in Figure 1B and Table 1, respectively. High Trop2 expression in cytoplasm was significantly correlated with tumor location (*p* = 0.046), lymph nodes metastasis (*p* = 0.027), and TNM stage (*p* = 0.031), while high Trop2 expression in stroma was remarkably associated with perineural invasion (*p* = 0.024), vascular invasion (*p* = 0.047), lymph nodes metastasis (*p* = 0.020) and TNM stage (*p* = 0.003).

**Table 1:** Association of Trop2 expression with clinical attributes of PC

Groups	No.	Trop2 expression in cytoplasm					Trop2 expression in stroma			
		Low or no expression (%)	High expression (%)	Pearson χ^2^	*p* value	No.	Low or no expression (%)	High expression (%)	Pearson χ^2^	*p* value
Age (years)										
≤ 60	65	26 (40.00)	39 (60.00)	0.757	0.384	57	33 (57.89)	24 (42.11)	0.614	0.433
> 60	99	33 (33.33)	66 (66.67)			84	43 (51.19)	41 (48.81)		
Insufficient data	25	16	9			14	8	6		
Gender										
Male	102	35 (34.31)	67 (65.69)	1.450	0.228	87	49 (56.32)	38 (43.68)	0.082	0.775
Female	74	32 (43.24)	42 (56.76)			63	34 (53.97)	29 (46.03)		
Insufficient data	13	8	5			5	1	4		
Tumor size (cm)										
≤ 2	15	6 (40.00)	9 (60.00)	0.139	0.933	14	10 (71.43)	4 (28.57)	1.404	0.496
2–3	35	14 (40.00)	21 (60.00)			28	15 (53.57)	13 (46.43)		
> 3	95	35 (36.84)	60 (63.16)			83	46 (55.42)	37 (44.58)		
Insufficient data	44	20	24			30	13	17		
Tumor location										
Head	97	33 (34.02)	64 (65.98)	6.151	0.046*	79	37 (46.84)	42 (53.16)	4.549	1.103
Body + Tail	49	18 (36.73)	31 (63.27)			45	30 (66.67)	15 (33.33)		
Insufficient data	43	24 (55.81)	19 (44.19)			31	17 (54.84)	14 (45.16)		
Differentiation										
Well + Moderate	149	58 (38.93)	91 (61.07)	0.168	0.682	123	65 (52.85)	58 (47.15)	0.436	0.509
Poor	40	17 (42.50)	23 (57.50)			32	19 (59.38)	13 (40.63)		
Perineural invasion										
Positive	64	23 (35.94)	41 (64.06)	0.236	0.627	54	27 (50.00)	27 (50.00)	5.114	0.024*
Negative	14	6 (42.86)	8 (57.14)			13	11 (84.62)	2 (15.38)		
Insufficient data	111	46	65			88	46	42		
Vascular invasion										
Positive	20	7 (35.00)	13 (65.00)	0.333	0.564	17	7 (41.18)	10 (58.82)	3.952	0.047*
Negative	47	20 (42.55)	27 (57.45)			42	29 (69.05)	13 (30.95)		
Insufficient data	122	48	74			96	48	48		
Lymph nodes metastasis										
Positive	40	10 (25.00)	30 (75.00)	4.908	0.027*	34	13 (38.24)	21 (61.76)	5.433	0.020*
Negative	90	41 (45.56)	49 (54.44)			79	49 (62.03)	30 (37.97)		
Insufficient data	59	24	35			42	22	20		
TNM stage										
Stage I	58	25 (43.10)	33 (56.90)	6.950	0.031*	56	40 (71.43)	16 (28.57)	11.340	0.003*
Stage II	31	12 (38.71)	19 (61.29)			26	14 (53.85)	12 (46.15)		
Stage III–IV	54	11 (20.37)	43 (79.63)			49	19 (38.78)	30 (61.22)		
Insufficient data	46	27	19			24	11	13		

* *p* < 0.05.

### Survival analysis

Both univariate and multivariate analyses consistently revealed that cytoplasm expression of Trop2 was the most significant predictor for poor survival in 189 patients with PC (*p* = 0.002 and 0.013, respectively) (Table 2). The Kaplan-Meier survival curves also demonstrated that PC patients with high Trop2 expression suffered a significantly shorter survival time (Figure 1C).

**Table 2:** Univariate and multivariate analysis of prognostic factors in PC for overall survival

Variable	Univariate analysis			Multivariate analysis		
	HR	*p* value	95% CI	HR	*p* value	95% CI
Trop2 expression in cytoplasm High versus Low or no	3.592	0.002*	1.605–8.036	3.337	0.013*	1.294–3.077
Trop2 expression in stroma High versus Low or	2.100	0.043*	1.024–4.306	1.401	0.401	0.638–3.077
Gender Female versus Male	1.392	0.368	0.677–2.863			
Age (years) ≤ 60 versus > 60	0.893	0.744	0.452–1.762			
Tumor size (cm) ≤ 2 versus 2–3 versus > 3	0.774	0.298	0.477–1.254			
Tumor location Head versus Body + Tail	0.666	0.095	0.414–1.072			
Differentiation						
Well + Moderate versus versus Poor	1.746	0.160	0.803–3.797			
Perineural invasion						
Positive versus Negative	0.575	0.593	0.076–4.365			
Vascular invasion						
Positive versus Negative	1.461	0.419	0.582–3.669			
Lymph nodes metastasis						
Positive versus Negative	2.133	0.038*	1.041–4.370	1.780	0.146	0.818–3.873
TNM stage Stage I versus Stage II versus Stage III–IV	1.295	0.235	0.846–1.982			

* *p* < 0.05.

### Characterization of Trop2Fab

**Figure 2:**
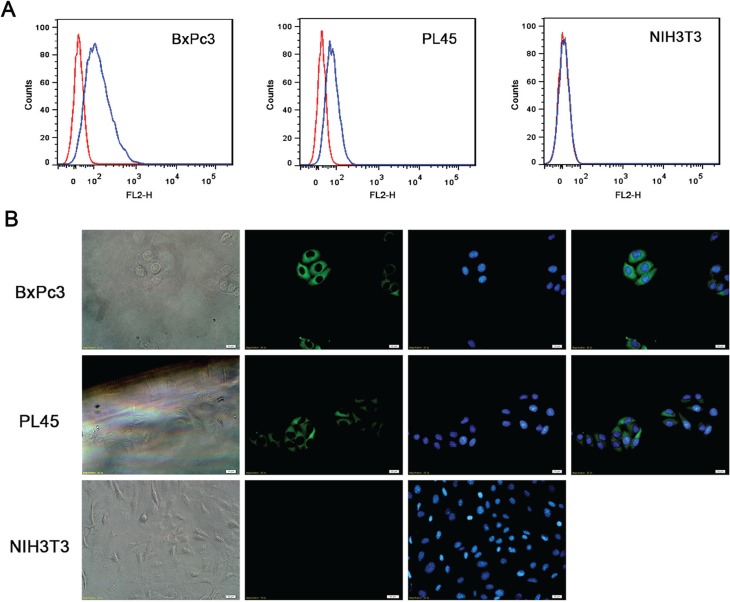
Trop2Fab reacts with Trop2-expressing PC cells (**A**) FACS analysis of BxPC3, PL45, and NIH3T3 cell lines. The fluorescent intensity differed significantly between Trop2-positive cells (BxPC3 and PL45) and Trop2-negative cells (NIH3T3). (**B**) Immunofluorescence assay showed that Trop2Fab could combine with BxPc3 and PL45 cells, and positive green signals were mainly located on cell surface, while NIH3T3 cells showed negative immunofluorescence signals. Blue signals were DAPI staining for cell nuclei.

The Trop2Fab was previously constructed in our laboratory [16] and was further characterized in this present study. In FACS analysis, the population of Trop2Fab-treated BxPc3 and PL45 cells was clearly separated from untreated cells by fluorescent intensity, with no observable difference between Trop2Fab-treated and untreated NIH3T3 cells (Figure 2A). In immunofluorescence assay, Trop2Fab was found to combine with BxPc3 and PL45 cells, and positive green signals were mainly detected around cell surface. In contrast, NIH3T3 cells exhibited negative signals (Figure 2B).

### Characterization of Trop2Fab-DOX

**Figure 3:**
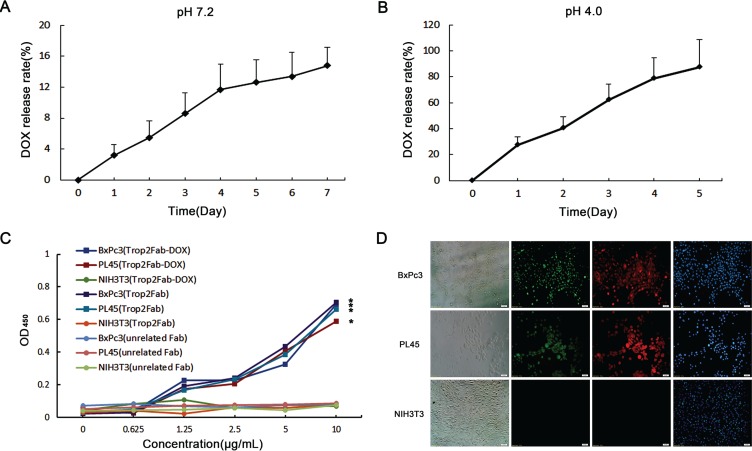
Characterization of Trop2Fab-DOX (**A**) The Trop2Fab-DOX was stable in pH 7.2 PBS. The amount of cumulated DOX release was about 15.0% over 7 days. (**B**) The DOX released from Trop2Fab-DOX quickly in pH 4.0 PBS, and almost 90.0% DOX was released within 5 days. (**C**) BxPC3, PL45 and NIH3T3 were incubated with Trop2Fab-DOX and Trop2Fab (0.625–10 μg/mL). Both Trop2Fab-DOX and Trop2Fab can react with BxPC3 and PL45 cells specifically, but not with NIH3T3 cells. Absorbance values were analyzed by Variance Analysis. *Significant difference of absorbance values in BxPC3 and PL45 cells compared with NIH3T3 cells. *p*< 0.05. (**D**) Binding efficacy of Trop2Fab-DOX was assessed by immunofluorescence observation. Both BxPC3 and PL45 cells presented green fluorescence after incubation with Trop2Fab-DOX. No signal was observed in NIH3T3 cells when incubated with Trop2Fab-DOX. Red signals were staining by commercial Trop2 antibody in PC cells and blue signals were DAPI staining for cell nuclei.

**Figure 4:**
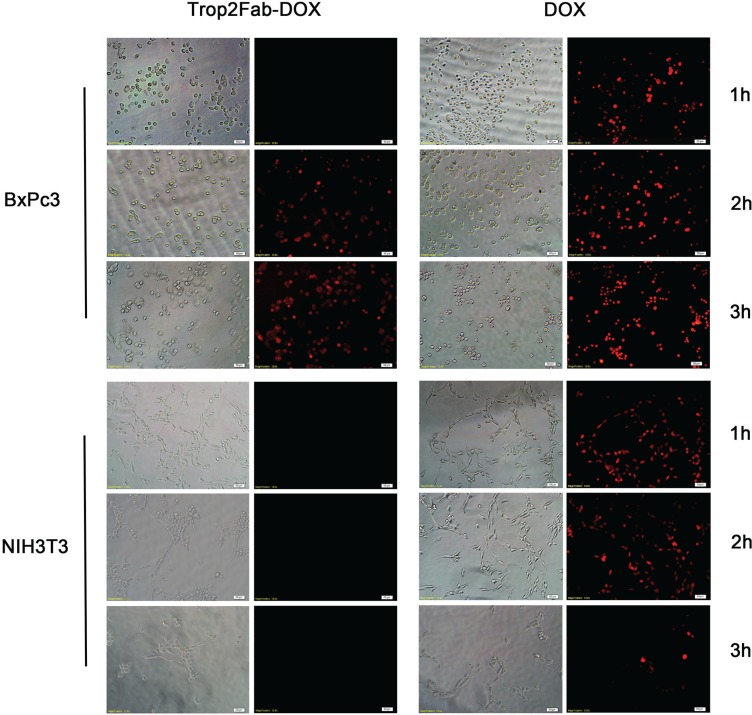
Detection of drug transport route by fluorescence microscopy BxPC3 and NIH3T3 cells were incubated with Trop2Fab-DOX and free DOX for 1 h, 2 h, and 3 h, respectively. For BxPC3 cells, the Trop2Fab-DOX was gradually distributed, and with prolonged time, the signal of Trop2Fab-DOX can be observed in the membrane, cytoplasm, perinuclear area and nucleus. In contrast, red fluorescent signals were quickly detected in the nuclei of cells after incubation with free DOX and the signals barely changed with prolonged time. For NIH3T3 cells, no red fluorescent signal was observed when incubated with Trop2Fab-DOX.

After the conjugation of Trop2Fab with DOX, the DOX release profiles were detected. As is shown in Figure 4A and 4B, the DOX release behavior from Trop2Fab-DOX was stable in pH 7.2 PBS, and the amount of cumulated DOX release reached nearly 15.0% over 7 days (Figure 3A). In comparison, the DOX was easier to release in pH 4.0 PBS than that in pH 7.2 PBS, and almost 90.0% DOX was released within 5 days (Figure 3B). It is mainly due to that the amide bond of Trop2Fab-DOX is solid in neutral solutions while vulnerable in acidic solutions. Moreover, as shown in cell ELISA analysis (Figure 3C), both Trop2Fab-DOX and Trop2Fab could specifically bind to BxPc3 and PL45 cells in a dose-dependent manner, while there was barely any binding to NIH3T3 cells. Absorbance values of Trop2Fab-DOX and Trop2Fab in Trop2-positive and -negative cells differed significantly. In immunofluorescence assay, Trop2Fab-DOX could specifically bind to Trop2-positive BxPc3 and PL45 cells. The cells treated with Trop2Fab-DOX exhibited green fluorescence around cell surface, while no signal was detected in NIH3T3 cells incubated with Trop2Fab-DOX (Figure 3D).

### Route of Trop2Fab-DOX transported within PC cells

To explore the process of DOX transportation by Trop2Fab and further compare the internalized capability of Trop2Fab-DOX and DOX, BxPc3 and NIH3T3 cells were incubated with Trop2Fab-DOX and DOX at different time points. With the prolongation of culture time, a growing amount of red fluorescence was observed in the membrane, cytoplasm and nuclei of BxPc3 cells treated with Trop2Fab-DOX, while the red fluorescence remained stable in the nuclei of BxPc3 cells treated with DOX. In contrast, NIH3T3 cells only exhibited red fluorescence in the nuclei when treated with DOX, while no staining was observed when treated with Trop2Fab-DOX (Figure 4).

### Trop2Fab-DOX inhibits tumor growth *in vitro*

**Figure 5:**
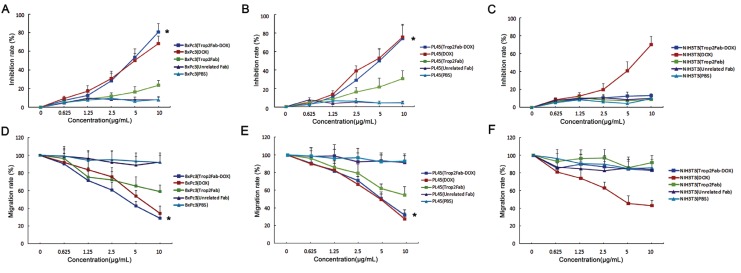
Trop2Fab-DOX inhibits the proliferation of PC cells *in vitro* (**A, B** and **C**) MTT assay showed a dose-dependent inhibitory effect of Trop2Fab-DOX (0.625–10 μg/mL) on the proliferation of BxPC3 and PL45 cells, while this inhibitory effect on NIH3T3 cells was nonsignificant. *Significant difference in BxPC3 and PL45 cell lines with Trop2Fab-DOX (10 μg/mL) compared with PBS treatment. *p* < 0.05. (**D, E** and **F**) Wound healing assay showed that the migration rate in BxPC3 and PL45 cells treated with Trop2Fab-DOX (10 μg/mL) was the lowest among all treated groups, the difference being statistically significant. While for NIH3T3 cells, wound closure rates in all treated groups showed no significant differences. *Significant difference in BxPC3 and PL45 cell lines with Trop2Fab-DOX (10 μg/mL) compared with PBS treatment. *p* < 0.05.

MTT assay and wound healing assay were performed to evaluate the inhibitory effect of Trop2Fab-DOX in Trop2-expressing PC cells. There were significant differences in inhibitory rate and migration rate of PC cells between Trop2Fab-DOX group and Trop2Fab group (*p* < 0.05) or PBS group (*p* < 0.05). For MTT assay, at 10 μg/mL of Trop2Fab-DOX after 72 h incubation, the growth of BxPc3 cells and PL45 cells were inhibited by 87.63% and 73.78%, respectively. Trop2Fab-DOX showed a potent and dose-dependent inhibitory effect on both PC cells and it also demonstrated more powerful tumor-inhibitory ability than DOX in BxPc3 cells. In contrast, Trop2Fab-DOX, Trop2Fab, unrelated Fab, and PBS exhibited modest or no inhibitory effects on NIH3T3 cells, although DOX still exerted an inhibitory ability (Figure 5A, 5B and 5C). For wound healing assay, the migration rate of BxPc3 cells and PL45 cells were decreased to 28.62% and 34.75% after incubation with 10 μg/mL of Trop2Fab-DOX for 72 h. Trop2Fab-DOX also demonstrated a dose-dependent inhibitory effect on cell migration. For NIH3T3 cells, the wound closure rates in Trop2Fab-DOX, Trop2Fab, unrelated Fab, and PBS showed no significant difference (Figure 5D, 5E and 5F). We also compared the IC_50_ of Trop2Fab-DOX and DOX indifferent PC cell lines. The fluorescence intensity of Trop2 was detected by FACS analysis to represent Trop2 expression levels of PC cells. As is shown in Table 3, according the concentration of effective-DOX, the ratio of IC_50_ (Trop2Fab-DOX) to IC_50_ (DOX) was 4.92 in BxPc3 cells with high Trop2 expression (98.12%) while about 4.43 in PL45 cells with moderate Trop2 expression (73.65%). For NIH3T3 cells, the ratio of IC_50_ can not be calculated because Trop2Fab-DOX barely showed inhibitory effects. The results indicated that the inhibitory effects of Trop2Fab-DOX are immunological specific in PC cells which have high Trop2 expression.

**Table 3:** The IC_50_ of DOX in PC cells treated with DOX or Trop2Fab-DOX with equivalent effective-concentration of DOX

Cell lines	DOX (μg/mL)		Trop2Fab-DOX (μg/mL)		Ratio of IC_50_	Trop2 expression*
	IC_50_	95% CI	IC_50_	95% CI		
BxPc3	0.86	0.376–0.993	4.23	2.659–7.575	4.92	98.12
PL45	0.35	0.168–0.697	1.55	0.437–4.669	4.43	73.65
NIH3T3	0.41	0.275–0.743	-			

* The Trop2 expression was illustrated as the value of fluorescence intensity by FACS. IC_50_(DOX). The IC_50_ value of DOX to cells after 48 h treatment. IC_50_(Trop2Fab-DOX): The IC_50_ value of Trop2Fab-DOX to cells after 48 h treatment. Ratio of IC_50_: IC_50_(Trop2Fab-DOX)/IC_50_(DOX).

### Trop2Fab-DOX inhibits tumor growth *in vivo*

**Figure 6:**
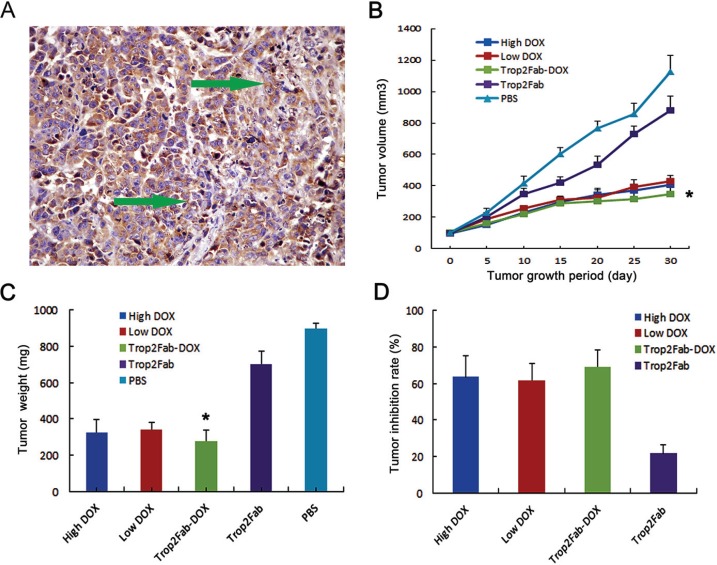
Trop2Fab-DOX inhibits the proliferation of PC cells *in vivo* (**A**) positive Trop2 expression in xenograft mice was confirmed by IHC analysis. Green arrow showed the positive staining of Trop2 in the cytoplasm in xenograft tumors. (**B**) Comparison of tumor volume of xenograft tumors in different treated groups. *p < 0.05 compared with control group. (**C**) Comparison of tumor weight of xenograft tumors in different treated groups. *p < 0.05 compared with control group. (**D**) Comparison of tumor-inhibitory rates in different treated groups. The inhibition rates in high concentration of DOX, low concentration of DOX, Trop2Fab-DOX and Trop2Fab were 63.69%, 61.84%, 69.16%, and 21.67%, respectively.

BxPC3 xenograft nude mice model was confirmed by IHC analysis for Trop2 expression (Figure 6A) and this model was employed to evaluate the tumor inhibitory effect of Trop2Fab-DOX *in vivo*. At 30th day, mean body weight of nude mice showed no substantial differences between all treated groups (data not shown). Three mice died (at 16th, 19th and 27th days) in high concentration DOX group, and two mice died (at 16th and 21st days) in low concentration DOX group. In contrast, no animal death was observed in other treated groups. As shown in Figure 6B and 6C, at 30th day the tumor volume and weight of PBS control group was 1123.8 (s.d.: 106.93) mm^3^ and 899.0 (s.d.: 30.43) mg, respectively. In contrast, the tumor size and weight were decreased to 428.8 (s.d.: 36.77) mm^3^ and 343.0 (s.d.: 36.57) mg in low concentration of DOX treated group, to 408.1 (s.d.: 29.41) mm^3^ and 326.5 (s.d.: 70.33) mg in high concentration of DOX treated group, to 346.6 (s.d.: 40.12) mm^3^ and 277.2 (s.d.: 60.69) mg in Trop2Fab-DOX treated group, and to 880.2 (s.d.: 90.44) mm^3^ and 704.2 (s.d.: 68.92) mg in Trop2Fab treated group, respectively. The inhibition rate of tumor growth in each treatment group is summarized in Figure 6D, and Trop2Fab-DOX was found to exert the most significant inhibitory effect on xenograft PC tumor growth.

## DISCUSSION

Although various therapeutic mono-clonal antibodies (mAbs) have been successfully applied in clinical practice, free antibodies targeting cell surface antigens that are expressed on tumors become less therapeutically effective by themselves or even though in combination with chemotherapeutic drugs [23, 24]. A wealth of evidence supports the claim that ADCs can enhance antitumor activity, reduce systemic toxicity and bring clinical benefits [25, 26]. The key factors for ADC development mainly include the selection of specific antigen, optimization of the kinetics, and efficacy of ADCs [27].

Trop2 has been identified as an oncogene for several human cancers [28–30] and a number of strategies targeting Trop2 have gained promising achievements [13, 31, 32]. What Trop2 functions in PC and whether the application of Trop2Fab in PC treatment is beneficial deserves further exploration.

We first observed a significantly higher level of Trop2 expression in PC tissues than in non-cancerous tissues by qPCR test and IHC analysis. Also Trop2 expression was identified to be closely correlated with certain malignant behaviors of PC patients. Further survival analysis demonstrated that the prognosis of PC patients with high Trop2 expression was critically worse than that of patients with low or no expression. Collective data on basis of previous research and the present study supported the notion of Trop2 as a appealing therapeutic target against cancer [13, 14, 28–30].

We previously stated that only high concentration of Trop2Fab can exert a tumor inhibitory effect [16], which clearly restricted its application. It was noted that RS7 antibody could rapidly internalize into target cells but barely had therapeutic activity in unconjugated form [33–35]. Echoing from this inspiration, we followed the rationale of ADCs and successfully constructed a conjugate of Trop2Fab-DOX, which can presumably improve the anti-tumor activity of Trop2Fab.

For ADCs, the linker is critical for maintaining the stability of the ADCs and releasing active drug in appropriate time and site [36, 37]. Trop2Fab-DOX is constructed by chemical synthesis, which contains an amide bond that is relatively stable in pH 7.2 PBS, while is able to release drug at pH 4.2. The data of DoX release test implies that this Trop2Fab-DOX does not release drug in human plasma under neutral pH environment, but allows DOX to be released from the antibody vector after pH change. Moreover, as evaluated by cell ELISA and immunofluorescence assay, the Trop2Fab-DOX conjugate could maintain the ability of recognizing Trop2 protein in cell membrane. Moreover, this conjugate had a particular way of releasing DOX. The observation of the process of drug transport in BxPc3 cells rendered us to quickly detect positive signals in nuclei only after cells incubated with DOX, and the reason may be that free DOX was delivered into cells by a passive diffusion style and bound to cell nucleus rapidly and stably from the beginning, and such binding barely changed regardless of elongation of incubation time. While as time went on, metabolic fluorescence was distributed on the surface, cytoplasm and nuclei of BxPc3 cells treated with Trop2Fab-DOX, implying the equilibrious transportation of DOX in BxPc3 cells. Meanwhile, the active DOX released from the conjugate and partitioned to the nuclei of BxPc3 cells was lower than the free DOX. Contrastingly, there was no detectable fluorescence in NIH3T3 cells treated with Trop2Fab-DOX, while high fluorescence in nucleus was observed in NIH3T3 cells treated with free DOX. These findings collectively suggest that DOX was rarely released from Trop2Fab-DOX conjugate in non-Trop2 expressing cells, which further proved the high selectivity and great specificity of Trop2Fab-DOX.

Although an anti-Trop-2 IgG-SN-38 conjugate was verified by Cardillo et al. to exert an antitumor effect [38], the use of the IgG antibody could not optimally recognize tumor marker since the existence of Fc fragments. In contrast, Fab antibody could reduce the false signals stemming from IgG antibody and improve the capability for identifying tumor antigen, such as Trop2 [39]. Moreover, Fab can facilitate trafficking of the antigens across cell membrane, rendering it more suitable to construct ADCs [40]. A recent study reported that a Tri-Fab bispecific antibody TF12 was sufficiently retained on cancer cell surface, which made it suitable for pre-targeted imaging and therapy of Trop2 expressing cancers [41].

In following tumor-inhibitory experiments *in vitro,* our results of MTT assay and wound healing assay demonstrated that Trop2Fab-DOX can effectively inhibit proliferation and migration of PC cells. Also, we assessed the antitumor effect of Trop2Fab-DOX *in vivo*. Although DOX was potent to inhibit tumor growth, its efficacy was lower than that of Trop2Fab-DOX in BxPc3 cells, suggesting the superiority of Trop2Fab-mediated specific tumor targeting. In addition, Trop2Fab-DOX exerted the highest tumor inhibition rate in PC xenograft mice and no animal death occurred in Trop2Fab-DOX treatment group. In comparison, there were three deaths in DOX treatment group. On this basis, it is reasonable to speculate that the lethal toxicity of DOX was reduced due to ADC conjugation.

It is also worth mentioning that the diversity of Trop2 expression in human cancers. For example, Trop2 expression was exclusively localized to the membrane in ovarian cancer cells while stroma cells were regularly negative [30]. In comparison, Trop2 expression could be observed in both cancer cells and stroma cells in colorectal cancer [42]. As for PC, Fong et al. detected homogeneous membranous expression of Trop2 in cancer cells [43]. While in this present study, Trop2 expression was observed in both cytoplasm and stroma of PC cells. Hence despite the promising results presented here, it should be stressed that the different levels of Trop2 expressing in PC may result in differential biologic efficacies of Trop2Fab-DOX.

In summary, our findings herein provide convincing evidence that Trop2 is a specific marker for PC, and a novel Trop2Fab-DOX ADC has a potent antitumor activity. Moreover, this Trop2Fab-DOX is effective both *in vitro* and *in vivo* against PC, and might serve as a promising target for cancer therapy that merits thorough preclinical researches in the future.

## MATERIALS AND METHODS

### PC tissue samples

Ten fresh PC tissues and the corresponding adjacent noncancerous tissues were obtained from the Department of Pathology, the Affiliated Cancer Hospital of Nanjing Medical University, Jiangsu Cancer Hospital. Simultaneously, 189 formalin-fixed, paraffin-embedded PC tissues and 87 non-cancerous tissues were obtained from patients undergoing surgical therapy for PC at the Affiliated Hospital of Nantong University between 2003 and 2010. Clinical data were collected from patient medical records. Tissue microarray (TMA) chips were generated using the manual Tissue Microarrayer System Quick Ray (UT06 UNITMA, Korea) in the Department of Pathology, the Affiliated Hospital of Nantong University. Survival time was calculated from the date of surgery to the date of death or last follow-up, whichever came first. Written informed consent was provided by all participants before participation in this study. All study protocols were reviewed and approved by the Human Research Ethics Committees of each local institution and all experimental methods were carried out in accordance with approved guidelines of Nantong University and Nanjing Medical University.

### Cell lines and reagents

Two pancreatic adenocarcinoma cell lines (BxPC3 and PL45) and a mouse fibroblast cell line NIT3T3 were purchased from the Cell Bank of Chinese Academy of Sciences (Shanghai, China). BxPC3 and PL45 are two Trop2-positive cell lines [44] and NIH3T3 is a Trop2-negative cell line. All cell lines were cultured in DMEM-H (Invitrogen, Carlsbad, CA, USA) supplemented with 10% fetal bovine serum (FBS) (Gibco), penicillin (100 U/mL) and streptomycin (100 lg/mL). Human anti-Trop2 Fab antibody (Trop2Fab) and a self-made anti-rabies virus Fab antibody (unrelated Fab) were prepared in our laboratory. The commercial anti-Trop2 antibody for IHC analysis in TMA was purchased from the Abcam Co., Ltd (Abcam, England). Doxorubicin (DOX) (2 mg/mL) was kindly provided by the Southeast University.

### Detection of Trop2 expression by qPCR and IHC analyses

For one-step quantitative real-time reverse transcription-polymerase chain reaction (qPCR) test, the primers for Trop2 were as follows: forward: 5′-TAT TAC CTG GAC GAG ATT CCC C-3′ and reverse: 5′- CCC CGA CTT TCT CCG GTT G-3′. Glyceraldehyde 3-phosphate dehydrogenase (GAPDH) was used as an internal control. The primers for GAPDH were as follows: forward: 5′-TGC ACC ACC AAC TGC TTA GC-3′ and reverse: 5′-CTC ATG ACC ACA GTC CAT GCC-3′. Total RNA extraction, amplification conditions and one-step qPCR procedure were described in detail in our previous reports [45–47]. For IHC analysis, TMA sections were incubated with a primary monoclonal mouse anti-Trop2-antibody (1:200, Abcam). The IHC evaluation of Trop2 was defined in our previous reports [48–54].

### FACS analysis and immunofluorescence assay for Trop2Fab

Both fluorescence-activated cell sorting (FACS) analysis and immunofluorescence assay were carried out on BxPC3, PL45, and NIH3T3 cell lines according to the methods that described in our previous researches [16, 22].

### Conjugation of Trop2Fab with DOX

**Figure 7:**
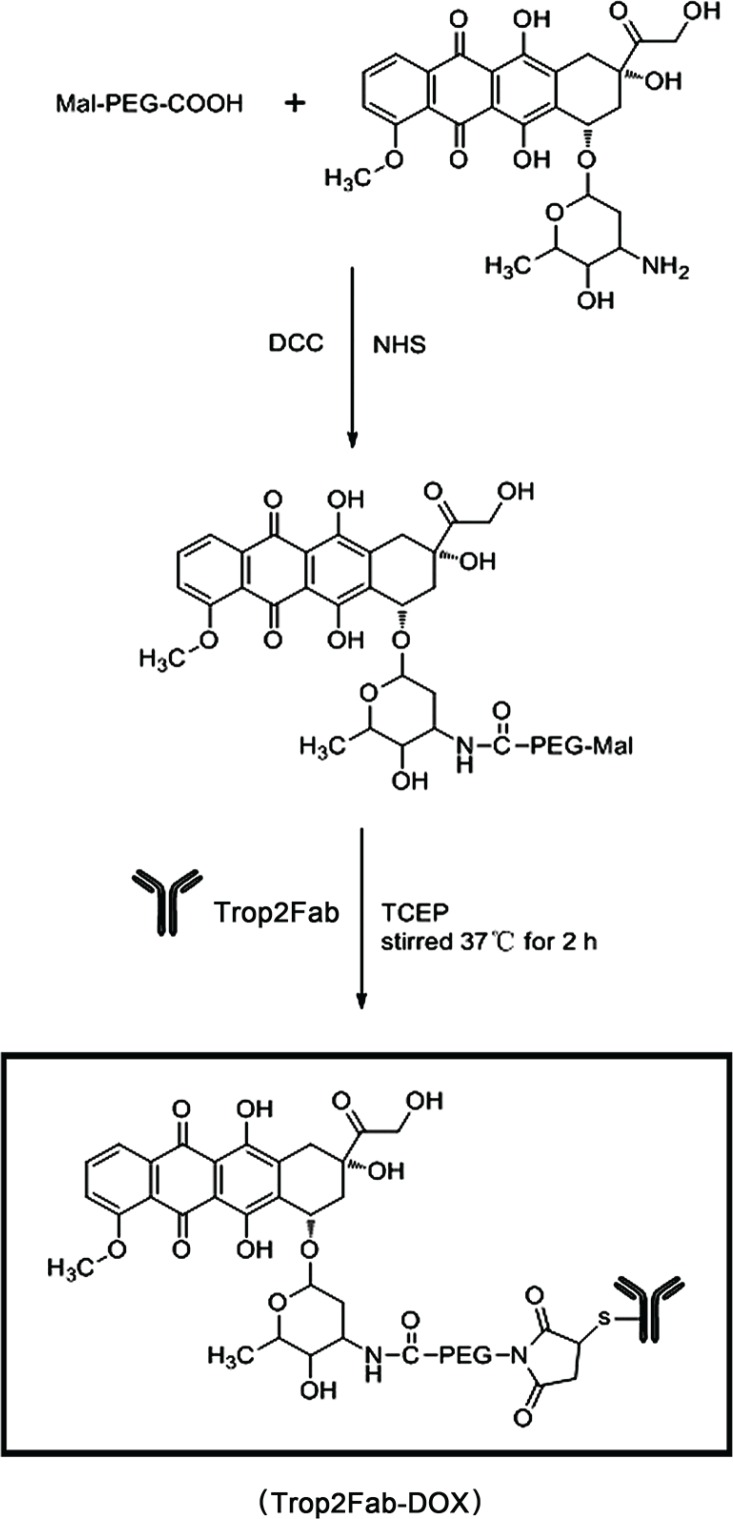
Conjugation of Trop2Fab fragment with DOX MAL-PEG-COOH was dissolved in dichloromethane to react with DOX. The reaction was activated by DCC/NHS to form the MAL-PEG-DOX and an amide bond was created. Then MAL-PEG-DOX was added to the solution of Trop2Fab and TCEP in PBS, stirred at 37°C for 2 h to construct Trop2 Fab-DOX and a thioether bond was established. Trop2Fab-DOX was marked by a black frame.

As shown in Figure 7, MAL-PEG-COOH was dissolved in dichloromethane to react with DOX under the activation by DCC/NHS to form the MAL-PEG-DOX. Then MAL-PEG-DOX was added to the solution of Trop2Fab and TCEP in PBS, stirred at 37°C for 2 h to construct Trop2 Fab-DOX.

### Detection of the release of DoX from Trop2 Fab-DOX

The release rate of DOX from Trop2 Fab-DOX was detected by a spectrophotometer. Briefly, the Trop2 Fab-DOX was suspended in 1 mL of PBS in a filter bag, which was then placed in 300 mL medium of PBS (pH 7.2 or pH 4.0) and shaken in an orbital shaker at 37°C. The 1 mL medium was acquired and sampled for DOX concentration, and further analyzed for the accumulated release rate of DOX from Trop2 Fab-DOX.

### Cell ELISA and immunofluorescence assay for Trop2Fab-DOX

For cell enzyme-linked immunosorbent assay (ELISA), BxPC3, PL45 and NIH3T3 cell lines were harvested in a logarithmic growth phase, and seeded on a 96 wells cell-culture plate at a cellular density of 10^4^ cells per mL. After incubating and washing, the plate was blocked with 1% non-fat milk for 2 h, followed by the addition of 50 μL Trop2Fab-DOX and Trop2Fab with the same gradient effective-antibody concentrations from 0.625 to 10 μg/mL in triplicate wells. Then a goat anti-human Fab coupled with horseradish peroxidase (HRP) antibody was added (1:2000). After washing, observation was executed with TMB solution at room temperature for 30 min and stopped by 1 M H_2_SO_4_. The optical absorbance was measured at 450 nm. For immunofluorescence assay, BxPC3 and PL45 cell lines were cultured in 6-well plates and immunofluorescence assay was performed according to the method described previously [16]. The unrelated Fab was used as a negative control in both assays.

### Route of Trop2Fab-DOX transported in PC cells

BxPC3 and NIH3T3 cell lines were seeded into a 96-well cell culture plate at a density of 6 × 10^3^ cells per well, and treated with Trop2Fab-DOX and DOX (10 μg/mL). The fluorescent signal of doxorubicin was examined by fluorescence microscopy after treatment for 1 h, 2 h and 3 h, respectively. The detailed protocol was described in our former studies [16, 22].

### MTT assay

After incubating BxPC3, PL45 and NIH3T3 cell lines with various concentrations of Trop2Fab-DOX, DOX, Trop2Fab and unrelated Fab (0.625–10 μg/mL) for 24 h, 48 h and 72 h, MTT assay was performed according to the methods described previously [55, 56]. PBS was used as a negative control.

### Wound healing assay

BxPC3, PL45 and NIH3T3 cell lines were treated with Trop2Fab-DOX, DOX, Trop2Fab and unrelated Fab with different concentrations (0.625–10 μg/mL) and wound healing assay was performed according to the method described previously [16]. PBS was used as a negative control.

### BxPC3 xenografts

Four-week-old BALB/c nude mice with a body weight of 18–20 g were purchased from the SLAC Laboratory Animal (Shanghai, China), and they were kept under specific pathogen-free conditions. For BxPC3 xenograft establishment, all mice were injected with 5 × 10^6^/mL BxPC3 cells subcutaneously in a volume of 0.1 mL into the flank. When tumors reached 100 mm^3^ after inoculation, the mice were randomly and evenly assigned to five groups (8 mice per group). Each mouse was injected intravenously on every two days with different drugs: group I: high concentration of DOX (2 mg/kg); group II: low concentration of DOX (1 mg/kg); group III: Trop2Fab-DOX (containing 2 mg/kg of equivalent DOX); group IV: Trop2Fab (6.52 mg/kg, the same antibody concentration as Trop2Fab-DOX); group V: PBS as a negative control. After xenograft transplantation, mice bearing tumors were observed and tumor size was measured and recorded once every 2–3 days. Tumor volume was estimated according to the method described previously [16]. At the end of the treatment period (30th day), all mice were killed, and tumors were excised to determine tumor weight and perform IHC analysis. The animal studies were conducted in accordance with the Public Health Service Policy and approved by the Animal Care and Use Committee of Nanjing Medical University.

### Statistical analysis

Trop2 expression was analyzed by the Wilcoxon signed-rank test. The significance of Trop2 expression on clinicopathological parameters of PC was analyzed by the χ^2^ test. Univariate and multivariate analyses were performed to identify the risk factors responsible for overall survival. The Kaplan-Meier method was used to evaluate the association between Trop2 expression and clinical outcomes in patients with PC. Quantitative data were expressed as means ± standard deviation (s.d.), and analyzed by Variance Analysis and SNK-*q* test. The *p* < 0.05 was considered statistically significant for a computed two-tailed probability. All the statistical analyses were completed with the SPSS version 18.0 (SPSS Inc., Chicago, IL, USA) and STATA version 12.0 (Stata Corporation, College Station, TX, USA).
